# Pathways of Coagulopathy and Inflammatory Response in SARS-CoV-2 Infection among Type 2 Diabetic Patients

**DOI:** 10.3390/ijms24054319

**Published:** 2023-02-21

**Authors:** Orsolya-Zsuzsa Akácsos-Szász, Sándor Pál, Kinga-Ilona Nyulas, Enikő Nemes-Nagy, Ana-Maria Fárr, Lóránd Dénes, Mónika Szilveszter, Erika-Gyöngyi Bán, Mariana Cornelia Tilinca, Zsuzsánna Simon-Szabó

**Affiliations:** 1 Doctoral School, Faculty of Medicine, George Emil Palade University of Medicine Pharmacy, Science, and Technology of Târgu Mureş, 540142 Târgu-Mureș, Romania; 2 Department of Transfusion Medicine, Medical School, University of Pécs, 7624 Pécs, Hungary; 3 Department of Chemistry and Medical Biochemistry, Faculty of Medicine in English, George Emil Palade University of Medicine, Pharmacy, Science, and Technology of Târgu Mureş, 540142 Târgu-Mureș, Romania; 4 Department of Pathophysiology, Faculty of Medicine, George Emil Palade University of Medicine, Pharmacy, Science, and Technology of Târgu Mureş, 540142 Târgu-Mureș, Romania; 5 Department of Anatomy, Faculty of Medicine, George Emil Palade University of Medicine, Pharmacy, Science, and Technology of Târgu Mureş, 540142 Târgu-Mureș, Romania; 6 Clinic of Plastic Surgery, Mureș County Emergency Hospital, 540136 Târgu Mureș, Romania; 7 Department of Pharmacology, Faculty of Medicine in English, George Emil Palade University of Medicine, Pharmacy, Science, and Technology of Târgu Mureş, 540142 Târgu-Mureș, Romania; 8 Department of Internal Medicine I, Faculty of Medicine in English, George Emil Palade University of Medicine, Pharmacy, Science, and Technology of Târgu Mureş, 540142 Târgu-Mureș, Romania

**Keywords:** COVID-19, thrombosis, coagulopathy, vasculopathy, inflammation, diabetes mellitus

## Abstract

Chronic inflammation and endothelium dysfunction are present in diabetic patients. COVID-19 has a high mortality rate in association with diabetes, partially due to the development of thromboembolic events in the context of coronavirus infection. The purpose of this review is to present the most important underlying pathomechanisms in the development of COVID-19-related coagulopathy in diabetic patients. The methodology consisted of data collection and synthesis from the recent scientific literature by accessing different databases (Cochrane, PubMed, Embase). The main results are the comprehensive and detailed presentation of the very complex interrelations between different factors and pathways involved in the development of arteriopathy and thrombosis in COVID-19-infected diabetic patients. Several genetic and metabolic factors influence the course of COVID-19 within the background of diabetes mellitus. Extensive knowledge of the underlying pathomechanisms of SARS-CoV-2-related vasculopathy and coagulopathy in diabetic subjects contributes to a better understanding of the manifestations in this highly vulnerable group of patients; thus, they can benefit from a modern, more efficient approach regarding diagnostic and therapeutic management.

## 1. Introduction

Diabetes mellitus (DM) is a major and common public health issue in both developed and developing countries, due to its high mortality rate and induced disabilities.

COVID-19 is caused by the SARS-CoV-2 virus, and since its appearance it became a public health problem due to its rapid spread, the severity of the symptoms, and increased mortality, causing a pandemic, with serious medical, social, and economic consequences globally [1,2,3].

Chronic inflammation, present in DM, enhances the synthesis of several cytokines. This chronic inflammatory state is preceded by a subclinical inflammatory response, represented by elevated IL-1β and IL-6 before the onset of T2DM [4]. Multiple studies reported during the pandemic that severe forms of COVID-19 are associated with elevated inflammatory markers, and comorbidities [5,6,7,8].

Endothelial dysfunction is also a consequence of DM and leads to micro- and macroangiopathy, and concomitantly to hypercoagulability [9].

Scientists had reported from the beginning of the pandemic the association of thrombosis and hypercoagulability with COVID-19, and the urgent need to understand the underlying mechanism for adequate management [10,11,12]. COVID-19-associated coagulopathy (CAC) is potentially lethal and can lead to disabilities [13,14,15,16]. To prevent severe complications and reduce the mortality of COVID-19 patients, targeted therapies for the associated pathologies are required.

The authors have undertaken to write a review that integrates the mechanisms of COVID-19 coagulopathy in patients with type 2 diabetes mellitus (T2DM). To synthesize the paper, a comprehensive literature search on PubMed, Embase, and Cochrane Library was performed, using the following keywords: “SARS-CoV-2”, “T2DM”, “COVID-19 and diabetes mellitus”, “coagulopathy and T2DM”, “mechanism”, “inflammation” “cytokine storm”, “gene polymorphism”, “COVID-19 and coagulopathy”, “hypercoagulability and endothelial dysfunction”, “role of MASP-2 and COVID-19 hypercoagulability”, “complement activation and COVID-19”. Open-access, full-text English language articles published between the 1st of January 2020 and the 2nd of December 2022 were accessed. The first search included clinical trials, meta-analyses, and randomized controlled clinical trials, and returned 1126 results. In the second step, we narrowed our search area by screening the articles using titles and abstracts, reducing the number of articles to 200. After eliminating duplicates, full-text analysis and further reduction occurred, and finally, 101 manuscripts were selected and integrated into this review, without taking into consideration the scientific impact or citation numbers of each article.

The authors aimed to assess the links between the altered molecular pathways of coagulation on the background of chronic low-grade inflammation of diabetes mellitus and the pathomechanisms of COVID-19-associated coagulopathy and extreme inflammatory response. The secondary goal was to identify possible mechanisms that may be responsible for the higher risk of severe COVID-19 progression in diabetic patients.

The originality of the article is derived from the multiple interactions presented, which are involved in the pathomechanism of COVID-19-related vasculopathy and coagulopathy in type 2 diabetic patients. Novel research results are included, based on the latest articles in scientific literature, and the connections between different pathways are presented in the text and on a complex, original diagram.

A limitation of the study is the lack of long-term experience in basic research related to COVID-19 mechanisms, taking into consideration the relatively recent occurrence of this special epidemiological situation of the coronavirus pandemic. Another limitation is that exclusively open-access articles were used and the authors used only articles written in English, so data that were published in other languages or not in open access were not included in this review.

## 2. The Pathophysiology of COVID-19 and T2DM Coagulopathy

### 2.1. The Pathways of Diabetic Endothelial Dysfunction

The most common form of diabetes is T2DM, a heterogeneous disorder, characterized by relative insulin deficiency, and insulin resistance in target tissues. Insulin resistance could be the key mechanism in the development of T2DM and other pathologies, such as hypertension, obesity, coronary artery disease, and metabolic syndrome [17]. The lack of insulin response is the result of the decrease of insulin receptors on the target cell’s surface. Some authors have reported that altered endothelial cell signaling and activation of redox regulated transcription factors are contributors as well [18,19]. Normally, insulin binding to its receptors activates two major signaling pathways: the phosphatidylinositol 3-kinase (PI3K)-dependent insulin signaling pathway and the mitogen-activated protein kinase (MAPK)-dependent insulin signaling pathway. PI3K is responsible for metabolic changes and is regulating glucose transporter type 4 (GLUT4) translocation in adipose cells, while MAPK regulates mitogenesis, growth, and differentiation [17]. Endothelial production of nitric oxide (NO) is regulated by a PI3K-dependent insulin signaling pathway, with a vasodilator effect, also enhancing glucose uptake of skeletal muscles [17]. It was also described that insulin stimulates endothelin-1 (ET-1) secretion via the MAPK signaling pathway, leading to vasoconstriction. In T2DM, the overproduction of advanced glycation end products (AGEs) and inflammatory cytokines contribute to the development of macroangiopathy, and its main form, atherosclerosis. It was also described that oxidative stress and excess production of reactive oxygen species (ROS) are the consequences of the activated major pathways involved in the development of diabetes- related complications: polyol pathway, protein kinase C (PKC) isoforms, excess formation of AGEs, increased expression of AGEs receptor and its activating ligands, and overactivity of the hexosamine pathway [20,21,22]. Hyperglycemia in T2DM is also responsible for endothelial dysfunction as the consequence of insulin resistance and excessive production of ROS [17]. Oxidative stress will lead to decreased antioxidant effect and excess synthesis of hydrogen peroxide anion, which directly deactivates NO, resulting in decreased NO activity [20]. ROS in excess can induce epigenetic changes. All these mechanisms can be the common links between the development of diabetes, chronic inflammatory response, and cardiovascular diseases (CVD). Cardiovascular complications are present in approximately 80% of T2DM patients [18].

Vascular complications of T2DM include macrovascular, microvascular, cerebrovascular lesions, and peripheral artery disease [18,23].

Macrovascular arteriopathy can affect the central and peripheral arteries, while microvascular diseases affect the small blood vessels in multiple organs, leading to chronic kidney disease (CKD), retinopathy, and the most common type, peripheral neuropathy [24].

The vascular endothelium secretes vasoactive substances to maintain vascular homeostasis by regulating vasoconstriction and vasodilation. Angiotensin II (AT-II), thromboxane A2, and ET-1 have vasoconstrictor effects, while prostaglandin I2 and NO are vasodilators under physiological conditions [20].

The homeostasis of vascular function, especially blood pressure and volume control, is under the regulation of the renin–angiotensin–aldosterone system (RAAS), but RAAS is also known to be involved in local tissue homeostasis, with anti-inflammatory, anticoagulant, antiproliferative, antifibrotic, and antiapoptotic effects on epithelial cells via the ACE2 activated Mas-receptor axis [25].

In T2DM, vascular homeostasis is disturbed by endothelial dysfunction, oxidative stress, platelet hyperreactivity, and inflammation [26], causing alteration in the physicochemical properties of the vascular wall, and enhance the development of atherosclerosis. All these events will aggravate thrombosis and hypercoagulability [27].

### 2.2. The Pathomechanism of Endothelial Dysfunction in COVID-19

SARS-CoV-2 infects human cells using the ACE2 receptor and a specific transmembrane serine protease 2 (TMPRSS2), for the priming of the spike protein [28].

ACE2 is expressed in various tissues and organs, including endothelium, lung, heart, intestine, kidney, pancreas, and on the epithelial cells of oral mucosa and the tongue [29]. Reportedly, in T2DM patients the ACE2 receptors are upregulated. It has been hypothesized by many that overexpression of ACE2 receptors in T2DM potentially increases the susceptibility to COVID-19 [30,31].

Once SARS-CoV-2 binds to ACE2 receptors and blocks their activity, the RAAS will be affected. Consequently, accumulation of angiotensin 2 (AngII) will occur, which triggers intracellular signaling pathways (caspase 3, p83 MAPK, ROS, cytochrome C) [32], and leads to vasoconstriction, increased oxidative stress, inflammation, cellular damage, and fibrosis. The regulation of RAAS is influenced by the interaction between ACE2 and bradykinin (BK). Normally, BK acts as a negative regulator of RAAS by dilating blood vessels via local NO release. BK is known for its anti-inflammatory and antioxidant properties and has a role in regulating cytokine production and blood vessel permeability. It also has a stimulating effect on plasminogen secretion and thrombus formation. In COVID-19, the internalization of ACE2 will enhance the activation of different types of BK receptors, leading to increased inflammation and local vascular hyperpermeability. Indirectly, it may activate the coagulation cascade through the resulting endothelial damage [33]. The activation of p83 MAPK can contribute to inflammation and oxidative stress, and the activation of caspase 3 can lead to cellular death [33]. ROS formation will induce oxidative damage to cells and tissues and will release cytochrome C from mitochondria, which can trigger the activation of apoptotic signaling pathways and contribute to cell death as well [34].

Nuclear factor kappa B (NF-κB) is a key molecule involved in the nuclear translocation and activation of controlled genes. Overactivation of NF-κB will lead to the extensive synthesis of proinflammatory mediators, uncontrolled inflammatory response, and eventually to cytochrome storm, as observed in COVID-19 patients [35].

After the endothelial infection by the SARS-CoV-2, von Willebrand factor (vWF) is released into the circulation. The vWF is stored in the Weibel–Palade bodies of the endothelial cells. Platelet aggregation initiated by the increased release of vWF [14] will generate a deposition of platelet-rich clots in the lung microcirculation. This event is the key mechanism leading to respiratory failure [36]. Hypercoagulability will be sustained because of the associated release of factor VIII [14], but it is also the consequence of the virus replication within the endothelial cells. The infection causes endothelial cell death and consequently, the endothelial damage will launch the procoagulant reaction [37].

CAC is characterized by clot formation in the lungs, and elevated D-dimer levels at an early stage of COVID-19, but after the systemic activation of the coagulation and the development of disseminated microthrombosis, multiple organs will be affected [38]. Post-mortem autopsy of severe COVID-19 patients found diffuse alveolar damage, and inflammatory infiltrations with hyaline membrane formation in the lung, but also inflammation of the myocardium, focal pancreatitis, axon injury, glomerular microthrombosis, macrophage accumulation in the brain, and lymphocyte infiltrations of the liver [39].

COVID-19 infection determines endothelial activation by angiopoietin-2, a mediator stored also in the Weibel–Palade body, which is released as well, showing elevated circulating levels in COVID-19 and an association with the induction of procoagulant and proinflammatory reactions [40].

### 2.3. Inflammatory Response in COVID-19

The development of inflammatory processes is a key pathological feature of SARS-CoV-2 infection. From the early beginning of the pandemic, several studies have suggested that massive inflammatory cytokines and chemokines are released in COVID-19 [41].

The innate immune system plays an important role, so proinflammatory cytokine production is a desired phase of the immune response against a pathogen. However, in some cases of COVID-19 infection, proinflammatory cytokine release and synthesis are rapidly overactivated, leading to multisystemic damage to the infected host. Interleukin (IL) 2 and 6 (IL-2, IL-6), tumor necrosis factor-alpha (TNF-α), interferon-gamma (IFN-γ), macrophage inflammatory protein (MIP), and monocyte chemoattractant protein 1 (MCP-1) are among many other cytokines that are present in seriously ill COVID-19 patients [42,43].

#### 2.3.1. From Cytokine Formation to Cytokine Storm

During inflammation, IL-6-induced tissue factor is released by macrophages [44].

IL-6 is a proinflammatory cytokine that can stimulate the release of other cytokines and activate immune cells, contributing to the overall systemic inflammation observed in severe COVID-19 cases [45].

AngII triggers NF-κB activation, leading to hyperinflammation, mostly through increased synthesis of IL-6 and IL-1b, and subsequently enhancing the transcription of proinflammatory cytokines. These interleukins presented extremely elevated levels in case of severe COVID-19 [46,47].

The exaggerated expression of IL-6 and IL-6 receptor in COVID-19 leads to endothelial cell hyperactivation and a large amount of tissue factor is released, both processes leading to infection-induced coagulopathy. This event plays an important role in thrombocytopenia, although the cytokine storm is the trigger of thrombocytosis. IL-6 is also participating in the production of some coagulation factors (fibrinogen, factor VIII). Acting on the endothelium, IL-6 enhances the synthesis of vascular endothelial growth factor (VEGF), leading to vascular hyperpermeability and hypotension [48]. Additionally, other cytokines, such as TNF-α, IFN-γ, and IL-1β, have also been implicated in the cytokine storm observed in COVID-19 patients and can contribute to increased inflammation and hypercoagulability.

IL-6 and IL-1α have the crucial role of linking inflammation with the coagulation system. During the proinflammatory phase, these cytokines are present on activated platelets, monocytes, and endothelial cells. IL-1α has the role of activating the inflammatory cascade in thrombo-inflammatory conditions, but is also a key element of thrombogenesis, through its granulocyte recruitment effect, prolongation of clot-lysis time, and increasing thrombocyte activity [43]. Combined with TNF, IL-1 is the most important mediator of endogenous coagulation cascade suppression [44].

The exact mechanisms and interplay between cytokines in diabetes with SARS-CoV-2 infection remains an area of active research.

#### 2.3.2. Complement Cascade Activation

The activation of the complement system following infection with SARS-CoV-2, as the main participant of innate immunity plays an important role in thrombotic events, combined with endothelium disturbances, thrombocytopenia, and bleeding, all representing risk factors of poor clinical outcome [34].

The literature describes three pathways of complement activation (host–antigen contact, antigen–antibody complex trigger, lectin pathway), all in the defense of the host, leading to the synthesis of C3 and derivatives and activation of plasma proteins [49]. The host–antigen contact will activate the first pathway, the activation of the second pathway is caused by antigen–antibody complexes, and the third one is activated by the lectin pathway, which will bind polysaccharides on antigen surfaces to host cells [50]. At this point, the virus will invade host cells that express the ACE2 receptor and damage them, causing a thrombotic–inflammatory response, which further activates the complement system [51]. The particularity of COVID-19 is related to the lectin pathway component, the mannose-associated serine protease 2 (MASP-2), with a key function of thrombin activation and fibrin mesh formation. Complement cascade participants dysregulate the endothelial cells, affecting the action of clotting cascade proteins [50].

In diabetic patients, the complement system, as an innate humoral defense, will become dysregulated, with the consequences of chronic low-grade inflammation and increased risk of infections [52].

Factor XII (FXIIa) activation has a trigger effect on complement complex C1. A further procoagulant effect of complement activation is the initiation of thrombocyte aggregation [53]. These pathomechanisms reveal a close relationship between complement and coagulation cascade, leading to the reciprocal up-regulation of both processes.

In COVID-19 the complement (C3 and C5) is the mediator for developing inflammation [54]. The terminal C5b-C9 complement activation leads to a release of C3b and C5b fragments, with a proinflammatory role [50]. C3b is involved in the opsonization process, marking the SARS-CoV-2, to be destroyed by immune cells. It is important as well for recruiting macrophages and neutrophils, which can release cytokines, signaling molecules that coordinate the immune response. This will induce prostaglandin and leukotriene synthesis, boosting further proinflammatory cytokine production. In some cases, the release of cytokines can become excessive, leading to an overactive immune response [54].

Normally, these processes occur with the purpose of self-defense of the host. However, uncontrolled complement activation results in exaggerated inflammation and systemic procoagulation status with the installation of disseminated intravascular coagulopathy and cellular damage [50].

In the later stages of the complement cascade activation, C5b is involved in the formation of the membrane attack complex (MAC). MAC formation has a role in direct cell lysis. In COVID-19 patients, activation of C5b and formation of the MAC can contribute to inflammation and tissue damage in the lungs and can induce the development of severe symptoms and complications [55,56,57].

### 2.4. The Cytotoxicity of Neutrophil Extracellular Trap

Neutrophil extracellular trap (NET) release is a mechanism of the innate immune response, as a result of the interaction with activated platelets. It occurs through the explosive intravascular destruction of neutrophils and the release of nucleic substances in the extracellular space, providing a source of extracellular histones with significant cytotoxicity [58]. With the ability to trigger inflammation and thrombosis, NETs release into the extracellular space oxidizes enzymes (NADPH oxidase, nitric oxide synthase) [44,59].

It has also been reported that NETs are among the main drivers of immune-thrombosis in severe COVID-19 cases [60]. Some authors hypothesize that SARS-CoV-2 can directly activate platelets through interaction with its surface spike protein [61], which triggers the release of platelet granules containing proinflammatory and procoagulant factors. Additionally, cytokine release as the result of the immune response to infected cells can also contribute to platelet activation. In COVID-19, platelets are activated and play a role in microvascular thrombosis, leading to serious complications such as acute respiratory distress syndrome (ARDS) and multi-organ failure (MOF) [62].

From the outbreak of the SARS-CoV-2 pandemic, Nicolai et al., among others, hypothesized that activated platelets might induce severe forms of NETosis in some COVID-19 patients with severe symptoms, leading to immune-thrombosis and higher mortality. The team superfused neutrophils isolated from healthy patients with platelet-rich plasma from severe COVID-19 patients and healthy subjects, revealing that the thrombocytes of severe COVID-19 patients adhered at a significantly increased rate to neutrophils compared to the controls [63].

The release of prothrombotic FXII, vWF, TF, and fibrinogen by NETosis will also lead to a procoagulant microenvironment. Circulating histones will also activate further platelets through their Toll-like receptors, resulting in clot formation [34].

NETosis is considered by a few authors to be a prothrombotic risk factor in COVID-19. Thus, a possible therapeutic option for thrombosis risk mitigation is proposedly NET inhibition using neutrophil elastase inhibitors and adenosine receptor agonists [64].

### 2.5. Hypercoagulability

The hypercoagulability present in T2DM will be enhanced by SARS-CoV-2′s binding to the ACE2 receptor and the receptor’s internalization will alter ACE2 functionality. Normally the enzyme binds to AngII, transforming it into angiotensin 1-7 (Ang1-7) peptide with anti-inflammatory effects. Ang1-7 binds and activates a MAS-related transmembrane G-protein coupled receptor (MRGPCR) [34], this reaction will assure anti-inflammatory, antioxidant, and antithrombotic effects. The downregulation of ACE2 receptors will alter RAAS leading to the above-mentioned hypercoagulability, but also hyper-inflammation, hypertension, hypertrophy, and apoptosis [33].

Moreover, platelet dysfunction can also lead to hypercoagulable states [49]. Platelet activation occurs through the initiation of angiotensin II type 1 receptor (AT1R) and its release of plasminogen activator inhibitor 1 (PAI-1). Platelets are also triggered by the altered ACE2R function [65]. Another important aspect is that platelets have MRGPCRs that modify thrombosis via NO release, and this also contributes to clot formation. This may be the explanation for the importance of platelet activation in COVID-19 coagulopathy [65].

**Figure 1:**
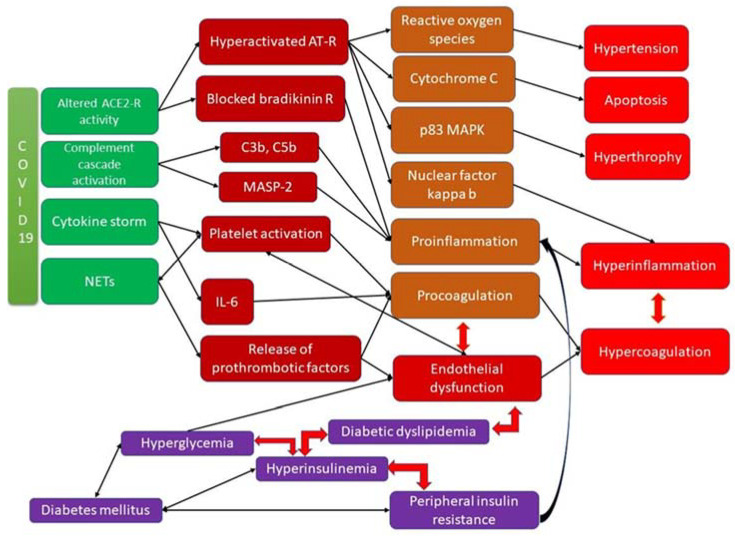
Coagulopathy in DM and COVID-19 infection and the underlying molecular mechanisms.

The interrelation between different factors and pathways involved in the development of arteriopathy and coagulopathy in diabetic patients is presented in Figure 1.

Hypercoagulability assessment using routine laboratory parameters in COVID-19 and T2DM is listed in Table 1.

In the clinical practice, for rapid assessment of CAC, a highly performant, point-of-care laboratory method was introduced.

Rotational thromboelastometry (ROTEM) is a point-of-care viscoelastic method for whole blood analysis, providing real-time information about clot formation, firmness, and fibrinolysis in severely ill patients and it is useful to identify a hypercoagulable state related to sepsis, COVID-19 [66,67,68]. Several ROTEM tests can be performed depending on the added substrate along with phospholipids and calcium. The extrinsic coagulation pathway is assessed using rotational thromboelastometry (EXTEM), which is initiated by adding a tissue factor. To assess fibrinolysis, fibrinogen rotational thromboelastometry (FIBTEM) is used, and it differs from EXTEM by using cytochalasin D, which inhibits the platelet cytoskeleton, so the whole clot formation depends on fibrinogen [66].

**Figure 2:**
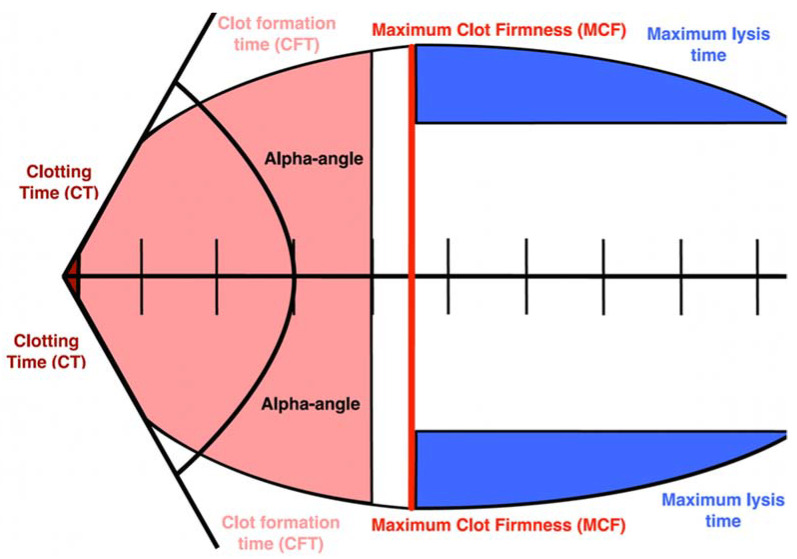
ROTEM parameters in COVID-19 Laboratory parameters and their association with the most frequent pathways in CAC are presented in Table 3.

Point-of-care hemostasis assessment in severe COVID-19 with ROTEM is represented in Table 2 and Figure 2, containing measured parameters, definitions, and levels in severe COVID-19.

The markers of the hypercoagulable state are as follows: shortened CT and higher MCF EXTEM and FIBTEM, shorter than normal EXTEM CFT, and higher alpha-angle [69]. Hypercoagulability could be assessed by ROTEM in 61% of cases [70].

ROTEM would be an appropriate point-of-care method for adequate assessment of coagulopathy, facilitating the work of clinicians to choose the most suitable therapy, applied individually based on the bedside results [66,67,68,70]. Furthermore, according to Schrick D. et al., ROTEM assays could also reveal platelet reactivity to antiaggregant therapy, and a lower reactivity was found to be associated with higher rates of lethal outcomes in severe COVID-19 cases [71].

### 2.6. The Importance of Genetic Background

The novel coronavirus breakout and pandemic have intensified the need for genetic investigations related to gene expression for a better understanding of the underlying pathomechanisms of SARS-CoV-2 and its genetic association with different diseases [72,73].

SARS-CoV-2 is a coronavirus of bat origin, causing a disease with various symptoms, from mild fever, cough, and sore throat in some patients or severe pneumonia, ARDS, and even septic shock or MOF in other individuals [74].

The intracellular pathogenicity of viruses makes them dependent on host cells, but it also suggests a virus–host protein–protein interaction (PPI). These PPIs have been the focus of recent analyses. The identification of the most common human proteins known to interact with coronavirus could provide a better understanding of the mechanism of COVID-19 and may suggest therapeutic strategies or drug combinations [75,76].

Using a network-based strategy, which incorporates gene expression profiling, gene ontologies, and PPI analysis, RNA-Seq scientists can identify molecular interactions between virus and host during the development of the infection and establish adequate treatment methods. RNA-Seq is a next-generation sequencing technology to measure gene expression with a high level of accuracy [76].

According to a study conducted by Islam et al. in 2021, cytokine activity and cytokine-mediated signaling pathways were predominant in COVID-19-associated T2DM. Similarly, TNF signaling pathway and cytokine–cytokine receptor interaction were found “enriched” [73].

The most frequently reported pathways were TNF and IL-17 signaling pathways, cytokine–cytokine receptor interactions, and photodynamic therapy-induced NF–κB survival. According to Ouyang et al., the TNF pathway is hyperactivated in severe COVID-19 [77]. On a background of T2DM, there is a direct involvement of TNF-α, through reduction of the insulin metabolism-related GLUT4 expression. Moreover, this phenomenon is also concomitant with insulin receptor inhibition by the serine phosphorylation of insulin receptor substrate-1 (IRS-1) [73]. IL-17, one of the principal triggers of cytokine storm in COVID-19, is released by the activation of the T-helper 17 lymphocyte (Th17) [55,78]. The IL-17 pathway has an insulin resistance-promoting effect, which is worsening the cytokine storm through AT1R excitation, leading to enhanced NO synthesis in diabetes [79,80].

CCL20 was found to have increased levels in the COVID-19-related cytokine storm, obesity, and insulin resistance. In pancreatic β-cells, CCL20 is regulated by NF–κB subunits. FOSL1 TF protein downregulates type I interferon (IFN-1) response, effective in the protection against viral infections [81], thus leading to viral susceptibility.

A study by Islam et al. identified 11 micro-RNAs (miRNAs) with shared pathogenetic potential between COVID-19 and diabetes: *miR-1-3p* and *miR-20a-5p* [73]. The *miR-34a-5p* miRNA decreases the antiapoptotic BCL2 protein, leading to increased glucose-mediated cardiomyocyte apoptosis [82]. Up-regulated *miR-34a-5p* is related to acute myocardial infarction causing heart failure [73]. In COVID-19, some prevalent miRNAs have been found to be associated with asthma (*miR-155-5p, miR-16-5p*) and other lung diseases (*let-7b-5p*). As a response to infection, *miR-146a* expression is induced by NF-κB. This will negatively affect IL-1 and TNF-α receptors so they attenuate inflammation. It has been shown that β-cell miRs are causing islet inflammation, leading to miR-146a-5p expression, which has down-regulated islet inflammation and beta-cell death by impairing NFκB and MAPK signaling [83]. The consequence of downregulated circulating miR-146 is hyper-inflammation in different organs [84]. Donyavi et al. have found that some miRNA can be used as biomarkers for the diagnosis of acute COVID-19 and to distinguish the acute phase from the post-acute form of COVID-19. The identified and suggested miRNAs as biomarkers were: *miR-29a-3p*, *miR-155-5p*, and *miR-146a-3p*. Thus, the connections between transcription factors and miRNAs with the pathogenesis of COVID-19 and diabetes may provide a better understanding of severe COVID-19 in diabetic patients [85,86].

The products of several genes involved in hyperglycemia, cytokine release, hormonal signals, receptor binding, and enzyme activities are interconnected and can influence the pathomechanism of COVID-19 and its complications in diabetic patients [87]. Genetic polymorphisms affecting ACE2 receptors, the cytochrome p450 system, or the cytoprotective heme oxygenase can complicate the treatment of COVID-19 by enhancing a proinflammatory and prooxidant state, increasing the cytokine storm and inducing a prothrombotic state [88].

Iessi et al. suggested that sex-related differences in the immune response may be transmitted via mitochondrial DNA, which could be responsible for the inferior function of male mitochondria and the observed reduced immune response in males [89]. The difference in immune response related to sex may also be explained by the bi-allelic expression [90] of X-linked genes encoding inflammatory mediators or receptors. Viveiros et al. suggested that due to the presence of the ACE2 gene on the X chromosome, which is considered as an X-gene escape, theoretically, women would have a double dose of ACE2, compensating for virus-mediated membrane ACE2 loss. However, ACE2 regulation is under the control of proteolytic cleavage and miRNAs; thus, the expression of ACE2 may not correlate with enzyme activity [90]. Gemmati et al. also hypothesized that women, due to the presence of two X-chromosomes, may have an advantage compared to men, based on their better adaptability to infectious diseases, such as COVID-19 [41].

The role of hyperglycemia in the development of cardiovascular diseases can be partially related to genetic background, although there is limited evidence. More than 150 loci showed association with coronary artery disease in the general population, and some of these loci (such as *9p21*) were clearly demonstrated to be involved in increased cardiovascular risk of diabetic patients, especially in case of poor glycemic control [91].

Certain pathomechanisms of diabetes are closely related to specific genetic and biochemical features, causing inflammation, fibrosis, apoptosis, and the release of ROS enhancing factors. Modified histone proteins, methylation of the genetic material, and modulation of microRNA expression are epigenetic changes which can regulate diabetic vascular complications despite adequate glucose control, or major signaling pathways in T2DM [18,92].

A bidirectional genetic interaction is described in the scientific literature between the human and viral genome during COVID-19. SARS-CoV-2 viral microRNAs can target different genes of the host organism (such as the *ADIPOQ* gene, playing an important role in metabolic syndrome) and human microRNAs were suggested to potentially target viral genes [93].

## 3. Summary

COVID-19 is characterized by coagulopathy and hemostatic imbalance.

Scientific evidence allows us to formulate the hypothesis that COVID-19-induced coagulopathy in T2DM develops more likely based on pre-existing vascular and metabolic disturbances through the pathomechanisms of the viral infection (intense cytokine release, endothelial dysfunction associated to infection, hyperinflammation, and hypercoagulable state).

Glycemic control will be a priority, not only for CVD protection, but also because ACE2 is present on pancreatic beta-cells as well, and pancreatic inflammation, induced by the cytokine storm, can lead to insulin resistance [94]. Several studies reported higher ACE2 expression in females, and decreasing ACE2 expression in elderly patients, which will be severely altered in the presence of DM. It was also reported that the cytokine storm has a repressing effect on ACE2 leading to severe outcomes [95].

It has been reported that chronic inflammation in T2DM will promote platelet activation leading to hypercoagulability. In T2DM the underlying condition, i.e., excessive level of proinflammatory cytokines and low level of anti-inflammatory cytokines, leading to an immunocompromised state, together with metabolic imbalance may be the explanation for the severe outcome of T2DM patients infected with SARS-CoV-2 [96]. The ROTEM method is a proper diagnostic tool for rapid evaluation of hypercoagulable state in COVID-19-related sepsis, which could be applied on a large scale.

Β-cell proliferation is observed in obese patients due to insulin resistance. It occurs as a compensatory response to nondiabetic obesity, when the organism is trying to counteract the insulin resistance by increasing insulin-secreting β-cells [97]. In some cases, the first sign of the onset of DM is in the form of ketoacidosis concomitant with COVID-19, leading to a severe outcome [98]. It has also been reported that those patients who are diabetics at the time of infection with SARS-CoV-2 have a better outcome than those with concomitantly installed DM and COVID-19. Certain studies also reported that patients with DM have a two-fold risk of intensive care unit hospitalization and a two–three-fold risk of hospital mortality than non-diabetic patients [99,100]. Several studies have shown that the infectivity of COVID-19 is not higher in patients with associated diabetes. The prevalence of diabetes in the COVID-19 patient population is not significantly different from the prevalence of diabetes in the general population [101].

Early diagnosis and identification of gene modifications that can influence the course of the disease is a desired aim to prevent further complications and to recognize risk levels for each patient.

The authors concluded that the mechanism of COVID-19, with the virus binding to the ACE2 receptor, might be different in patients in accordance with the individual genetic background or developed susceptibility.

We consider that human genetics plays an important role (inherited predispositions) in COVID-19 management, due to the possibility of identification of gene modifications that contribute to poor prognosis, even more, when pre-existing comorbidities and acquired risk conditions are present.

Comprehensive knowledge of the underlying pathomechanisms of coagulopathy and inflammatory response in diabetes mellitus contributes to a better understanding of the manifestations of angiopathy in this very vulnerable group of patients; thus, they can benefit from a modern, more efficient management.

## Figures and Tables

**Table 1:** Laboratory findings of the most assessed parameters in severe COVID-19 and type 2 diabetic patients [59].

Laboratory Parameters	Severe COVID-19 Patient	Type 2 Diabetic Patient
Hemoglobin	Anemia—moderate to severe	Normally unchanged or mild anemia
Platelets	Thrombocytopenia	Normal
Albumin	Hypoalbuminemia	Normal
ALT	Increased	May be increased due to comorbidities (fatty liver disease)
LDH	Increased	May be mildly enhanced due to increased apoptosis
Troponin I	Greater than 28 pg/mL	May be mildly increased due to enhanced apoptosis
D-dimer	Moderately increased	Slightly increased
Prothrombin time	Prolonged	Normal
Activated partial thromboplastin time	Prolonged	Normal
Ferritin	Greater than 300 μg/L	Normal or decreased

**Table 2:** Results of rotational thromboelastometry (ROTEM) in severe COVID-19 patients.

Parameter, UNIT	Definition	Reference Range	Levels in Severe COVID-19
Clotting time (CT), seconds	The time between the beginning of the coagulation and the increase in the amplitude of the thromboelastogram by at least 2 mm	EXTEM: 38–65 s FIBTEM: 55–87 s	EXTEM: 59 (32–128) mm FIBTEM: 66 (36–178) mm
Clot Formation Time (CFT), seconds	Time from the increase in the amplitude of thromboelastogram from 2 to 20 mm	EXTEM: 42–93 s	EXTEM: 47 (27–157)
Maximum Clot Firmness (MCF), millimeters	Maximum amplitude reached on the thromboelastogram	EXTEM: 53–68 mm FIBTEM: 9–27 mm	EXTEM: 65 (4–74) mm FIBTEM: 28 (9–42) mm
Alpha angle, angle	The slope of the tangent at 2 mm amplitude	EXTEM: 63–83°	EXTEM: 78 (68–83)°
Maximum lysis (ML), percent	The measure of fibrinolysis	EXTEM: 1–12%	EXTEM: 2 (1–13)%

Notes: EXTEM—extrinsically activated test, that is performed by the addition of tissue factor to the sample. FIBTEM—fibrin-based extrinsically activated test, performed with the addition of tissue factor and platelet inhibitor cytochalasin D. CT—clotting time. MCF—maximum clot firmness. CFT—clot formation time.

**Table 3:** Laboratory findings associated with thrombosis in intensive care unit COVID-19 patients [34,45,59].

Coagulation Biomarkers	Platelet Activation Biomarkers	Inflammation Biomarkers
D-dimer—increased	Thromboxane B2—increased levels associated to thrombosis and higher mortality	Extreme CRP levels—higher in patients with thrombosis compared to those without this complication
Fibrinogen—either increased or decreased	P-selectin- increased levels associated to thrombosis and higher mortality	Procalcitonin—higher in patients with thrombosis compared to those without this complication
Degradation products of fibrin/fibrinogen Increased values	Soluble CD40 ligand—increased levels associated to thrombosis and higher mortality	Erythrocyte sedimentation rate—high levels, higher in patients with thrombosis compared to those without this complication
Von Willebrand Factor Increased values	Mean platelet volume—increased levels associated to thrombosis and higher mortality	Ferritin—higher in patients with thrombosis compared to those without this complication
Prothrombin time, activated partial thromboplastin time—prolonged by 3 s (PT) and by 5 s (APTT)		
Platelet count—thrombocytosis		

## Data Availability

Not applicable.
